# From stigma to support: the mediating role of sympathy between nurses’ perceived stigma and helping behavior tendency for alcohol use disorder

**DOI:** 10.3389/fpsyt.2026.1811126

**Published:** 2026-04-22

**Authors:** Shufen Wang, Yanhua Qu, Qingyan Yang, Lei Lei Wang, Jing Shao

**Affiliations:** 1 Nursing Department, Beijing Huilongguan Hospital, Capital Medical University, Beijing, China; 2 Addiction Medicine Center, Beijing Huilongguan Hospital, Capital Medical University, Beijing, China; 3 Sleep Medicine Center, Beijing Huilongguan Hospital, Capital Medical University, Beijing, China

**Keywords:** alcohol use disorders, helping behavior tendencies, mediation analysis, nurses, stigma, sympathy

## Abstract

**Background:**

The detrimental effect of stigma on healthcare for individuals with alcohol use disorders (AUDs) is well-established, often resulting in social distance and diminished helping behavior tendencies. However, contemporary neuroscience reconceptualizes addiction as a brain disease, potentially altering emotional responses to stigma. This study examines a seemingly paradoxical possibility: that under specific conditions, perceived stigma is primarily associated with sympathy (rather than anger or fear), which in turn is linked to helping behavior tendencies among nurses.

**Methods:**

A cross-sectional survey was administered to 348 clinical nurses from tertiary hospitals in China. Participants completed standardized scales assessing perceived stigma of patients with AUDs, causal attributions, emotional responses (including sympathy, anger, and fear), and helping behavior tendencies. Data were analyzed using Pearson correlation and mediation analysis (PROCESS macro, Model 4) with 5,000 bootstrap samples to test the mediating role of sympathy.

**Results:**

Perceived stigma showed a significant positive correlation with sympathy (r= .160, p<.05), which was in turn positively correlated with helping behavior tendencies (r= .269, p<.05). Critically, mediation analysis revealed that sympathy fully mediated the relationship between perceived stigma and helping behavior tendencies. The standardized indirect effect was significant (β= 0.15, 95% CI [0.08, 0.23]), accounting for the total observed relationship, as the direct effect was non-significant. Additionally, compared to non-psychiatric nurses, psychiatric nurses perceived patients as significantly less dangerous and reported lower levels of fear and anger, along with a stronger intention to help and a lower tendency to avoid patients.

**Conclusion:**

Challenging conventional perspectives, this study supports a dual-pathway model in which perceived stigma can indirectly associated with professional helping behavior tendencies through the elicitation of sympathy. While other emotions like anger and fear were also measured, the findings highlight the pivotal role of cognitive-affective processes, shaped by neurobiological understandings of addiction, in determining nursing care. Specifically, sympathy, but not anger or fear, was found to mediate the stigma-helping relationship. Enhancing neuroscience-informed education and targeted empathy training, particularly for general nurses, could transform stigmatizing attitudes into supportive care, ultimately improving outcomes for patients with AUDs.

## 1 Introduction

The pervasive stigma surrounding alcohol use disorders (AUDs) constitutes a significant global barrier to effective treatment and recovery, adversely affecting patient engagement with healthcare services and their long-term outcomes (1–3).Within healthcare settings, nurses serve as primary caregivers, and their perceptions and behaviors are critically shaped by this stigma, which can undermine therapeutic relationships, communication quality, and ultimately their willingness to provide comprehensive care (4, 5). Consequently, understanding the determinants of nurses’ helping behavior tendencies toward this patient population is essential for improving care delivery and patient prognosis.

Helping behavior, defined as voluntary actions intended to benefit or support patients, is a fundamental component of quality nursing care (6). Its diminishment directly compromises therapeutic engagement and recovery. Extant research has consistently identified stigma as a pivotal social determinant that negatively influences health professionals’ attitudes and behaviors (7, 8). Dominant theoretical frameworks, notably Weiner’s attribution theory (9), posit that stigma rooted in perceptions of personal failure and controllability typically evokes negative emotions such as anger or blame, leading to avoidance and reduced helping; this represents a well-established detrimental pathway (10).

In contrast, the contemporary neurobiological model of addiction reconceptualizes it as a chronic brain disorder involving specific neuroadaptations, shifting the focus from volitional failure to underlying brain dysfunction (11, 12). This paradigm shift suggests an alternative cognitive-affective pathway: when healthcare providers attribute addictive behaviors to neurobiological causes rather than solely to personal choice, it may mitigate punitive judgments and elicit more compassionate emotional responses, such as sympathy (13). Sympathy, characterized by feelings of concern for another’s welfare and a motivation to alleviate suffering, is a recognized precursor to prosocial and helping behavior tendencies (14, 15). This presents a theoretically plausible yet underexplored paradox: under specific conditions, perceived stigma (i.e., nurses’ awareness of societal stereotypes and discrimination against AUD patients) might be indirectly related to helping behavior via sympathy.

Despite its potential significance, the specific psychological mechanism linking nurses’ perceived stigma of AUD patients to their helping behavior remains inadequately understood. Prior studies have predominantly focused on the direct negative consequences of stigma or examined broad constructs like general empathy (16, 17). The precise mediating role of discrete emotional responses, particularly sympathy arising from a neurobiological attributional shift, has not been empirically examined within the nursing context. Furthermore, contextual factors such as specialized training may moderate this relationship. Nurses in psychiatric or addiction specialties, who are often systematically trained in the biopsychosocial and neurobiological models of addiction, may demonstrate different patterns of emotional and behavior responses compared to general nurses (18, 19). Investigating this potential subgroup difference can yield critical insights for developing targeted interventions.

In this study, ‘perceived stigma’ specifically refers to nurses’ awareness of the negative stereotypes and discrimination that the public holds toward individuals with AUDs (i.e., perceived societal stigma), rather than their personal stigmatizing attitudes.

Building on this definition, the neurobiological model provides a critical lens through which this commonly perceived societal stigma may be appraised. By reconceptualizing addiction as a brain disease, this model directly challenges the attributions of personal responsibility and controllability that, according to Weiner’s theory, underpin stigma and lead to punitive emotions like anger. Consequently, when nurses are aware of societal stigma but interpret the patient’s condition through a neurobiological framework, their cognitive-affective response may pivot from blame to sympathy. This theoretical integration suggests that “perceived societal stigma” is not merely a negative backdrop but can serve as a catalyst that, when processed with a disease-based attribution, triggers a distinct emotional pathway (sympathy) leading to helping. This positions perceived stigma as a pivotal and ecologically valid starting point for examining divergent emotional and behavioral outcomes in nursing care.

While nurses may experience a range of emotions (e.g., anger, fear) in response to stigma, the present study focuses on sympathy as the hypothesized key mediator, based on the neurobiological attributional pathway. The roles of anger and fear are examined for comparative purposes, consistent with the traditional stigma-avoidance pathway outlined by attribution theory.

Therefore, integrating insights from social psychology and neuroscience, this study aims to address this gap by investigating a dual-pathway model in the context of nursing care for AUDs. Specifically, it seeks to: (1) test the hypothesis that sympathy mediates the relationship between perceived stigma and helping behavior tendencies, and (2) explore differences in perceived stigma, emotional responses, and behavioral tendencies between psychiatric and non-psychiatric nurses. Based on the theoretical framework and research objectives, the following hypotheses are proposed:

H1: perceived stigma of patients with AUDs will be positively correlated with nurse’ sympathy.
H2: Sympathy will be positively correlated with nurses’ helping behavioral tendencies toward patients with AUDs.
H3: Sympathy will mediate the relationship between perceived stigma and helping behavior tendencies.
H4: Psychiatric nurses will report lower levels of perceived stigma, fear, and anger, and higher levels of sympathy and helping tendency compared to non-psychiatric nurses.

The findings are expected to contribute to a more nuanced theoretical understanding of stigma’s operational pathways in healthcare and provide empirical evidence to inform the development of targeted, neuroscience-informed educational strategies aimed at improving care for individuals with AUDs.

## 2 Materials and methods

### 2.1 Study design and participants

This study employed a cross-sectional survey design. The study initially engaged the nursing department directors of 35 healthcare institutions across China. The target population comprised registered nurses actively engaged in direct patient care from these institutions. A convenience sampling method was employed. Participants were required to meet the following inclusion criteria: (1) possession of a valid nursing license, (2) current involvement in direct patient care, and (3) provision of informed consent. Exclusion criteria included: (1) intern or training nurses, and (2) individuals on extended leave during the data collection period.

### 2.2 Sample size determination

The minimum required sample size was determined *a priori* using G*Power 3.1 software. Based on Cohen’s guidelines for multiple regression with a medium effect size (f² = 0.15), an alpha level of 0.05, and a statistical power of 0.95, the calculation yielded a minimum sample of 267 participants. To account for potential missing data and non-response, an additional 20% buffer was incorporated, resulting in a target sample size of 334 participants. This approach ensured adequate statistical power for the planned analyses.

### 2.3 Measures and instruments

#### 2.3.1 General and professional characteristics

A comprehensive demographic questionnaire collected information on age, years of work experience, gender, education level, marital status, departmental affiliation (psychiatric vs. non-psychiatric), and professional title. These variables were used for sample description and as controls in subsequent analyses.

Perceived Stigma Scale for Patients with Substance Use Disorders (PSPS)

The Chinese version of the 9-item PSPS was used to assess nurses’ perceptions of societal stigma toward individuals with AUDs (20), that is, their judgment of how the general public views and treats such patients, not their personal agreement with those view. This scale was originally adapted from the Perceived Stigma of Addiction Scale (21) and has been validated in Chinese patient populations. To measure perceived societal (rather than personal) stigma, the items and response anchors were presented to nurse participants as inquiries about the beliefs and actions of “most people” or “society” (e.g., “Most people would…”), thereby capturing their appraisal of public attitudes. To precisely match the research context, the wording in all items was specified to refer to “AUDs”. In addition to good internal consistency (Cronbach’s α = 0.82 in this study), the use of this scale in a nurse sample is supported by its theoretically coherent pattern of correlations: PSPS scores showed a small but significant positive correlation with sympathy (r = .160, p <.05, see **Table 1**), aligning with the premise that awareness of societal stigma may, under certain cognitive appraisals, relate to sympathetic concern. While this provides preliminary evidence of construct validity, further validation of the scale’s factor structure in healthcare professional samples is warranted.

**Table 1:** Bivariate correlations among all measured variables (N = 348).

Variable	1	2	3	4	5	6	7	8	9	10	11	12
1. Perceived Stigma	1											
2. Attribution (Total)	.028	1										
3. Sympathy	.160*	.416*	1									
4. Dangerousness	-.032	.709*	.137*	1								
5. Fear	-.059	.712*	.134*	.641*	1							
6.Personal Responsibility	-.088	.624*	.085	.483*	.460*	1						
7. Anger	.016	.670*	.204*	.404*	.496*	.356*	1					
8.Segregation (Isolation)	-.021	.629*	.038	.391*	.352*	.348*	.342*	1				
9.Perceived Treatability	.064	.596*	.169*	.378*	.279*	.291*	.222*	.351*	1			
10. Helping Behavior Tendencies	.138*	.379*	.269*	.109	.104	.033	.258*	.114	.305*	1		
11. Avoidance	-.108	.415*	-.049	.294*	.341*	.350*	.229*	.258*	.105	-.295*	1	
12. Coercion	.046	.679*	.162*	.464*	.372*	.433*	.277*	.487*	.504*	.257*	.153*	1

PSPS, Perceived Stigma Scale for Patients with Substance Use Disorders; p<.05.

#### 2.3.2 Attribution questionnaire

A modified version of the Attribution Questionnaire (AQ) developed by Corrigan et al. (22) was employed to measure causal attributions, emotional responses, and behavioral tendencies toward a person with AUDs. This questionnaire has been widely used to assess stereotypes, attributions, and emotional reactions toward individuals with mental illness. The original AQ developed by Corrigan et al. (22) employs a vignette describing a male with schizophrenia. To ensure high relevance to the current research context, we adapted this vignette to describe a 32-year-old male (“David”) who had been repeatedly hospitalized due to AUDs, while retaining the original scale’s structure for measuring attributions, emotional responses, and behavioral tendencies. This vignette presents a hypothetical scenario and is not based on a real person. The AQ comprised 12 items measuring three key dimensions: (1) causal attributions (e.g., “To what extent is David responsible for his current condition?”), (2) emotional responses (sympathy, anger, fear), and (3) behavioral tendencies (helping, avoidance, coercion). All items were rated on a 9-point Likert scale ranging from 1 (not at all) to 9 (very much). For the purpose of this study, the sympathy subscale (e.g., “I feel sympathy for David”) and the helping behavior tendencies subscale (e.g., “I would be willing to help David”) were the primary focus of analysis. The overall AQ demonstrated good reliability in the current sample (Cronbach’s α = 0.79). The internal consistency for the specific subscales was also acceptable (sympathy α = 0.84; helping behavior tendencies α = 0.78).

#### 2.3.3 Demographic and professional characteristics

A self-designed questionnaire collected information on participants’ age, gender, years of work experience, education level, marital status, departmental affiliation (psychiatric vs. non-psychiatric) was categorized based on the participant’s current primary workplace at the time of data collection. These variables were used for sample characterization and as statistical controls in the analysis.

The survey instruments used in this study are provided in the **Supplementary Material**.

### 2.4 Data collection procedures

Data collection was carried out between January and June 2023. Anonymous self-administered questionnaires were distributed online via the Wenjuanxing platform. Trained research assistants coordinated with the nursing department managers of the participating institutions. The anonymous survey link was distributed by these managers to eligible nurses via the hospitals’ internal communication channels. Before accessing the questionnaire, all potential participants were presented with a detailed information sheet outlining the study’s purpose, procedures, and their rights. Electronic informed consent was obtained from each individual prior to proceeding. Participants completed the questionnaires in private settings to ensure confidentiality and minimize potential social desirability bias. To prevent duplicate submissions, each device’s IP address was restricted to a single response. A systematic monitoring process was implemented to track the return rate and address participant queries in real time. A total of 359 participants initially submitted the survey. After data cleaning, which involved excluding 11 participants who either completed the survey in less than 2 minutes or provided patterned/inconsistent responses, the final analytic sample comprised 348 nurses.

### 2.5 Statistical analyses

Data analysis was performed using IBM SPSS Statistics (Version 26.0) and the PROCESS macro (Version 4.1) for SPSS. First, descriptive statistics, including frequencies, percentages, means, and standard deviations, were computed to characterize the sample demographics and the distributions of the main study variables (perceived stigma, sympathy, and helping behavior tendencies). Second, Pearson’s correlation analysis was conducted to examine the bivariate relationships among these key variables. Third, independent samples t-tests were employed to compare the scores of psychiatric nurses and non-psychiatric nurses on the main variables, testing Hypothesis H4. Finally, to test the hypothesized mediation model (H3), Hayes’ PROCESS Macro (Model 4) was used with 5,000 bootstrap samples to obtain robust, bias-corrected confidence intervals (CIs) for the indirect effects. This number of resamples is recommended to ensure the stability and accuracy of the CIs (23). In this model, perceived stigma was specified as the independent variable (X), sympathy as the mediator (M), and helping behavior tendencies as the dependent variable (Y). The significance of the direct and indirect (mediation) effects was determined by examining the 95% bias-corrected confidence intervals (CI); an effect was considered statistically significant if its CI did not include zero. The significance level for all inferential tests was set at α= .05.

### 2.6 Ethical considerations

This study was conducted in accordance with the ethical principles outlined in the Declaration of Helsinki. The research protocol, including all procedures and materials, was reviewed and granted formal approval by the Institutional Ethics Committee (Approval Code: 2022-07-KE). Prior to their participation, all eligible nurses were provided with a comprehensive information sheet detailing the study’s objectives, procedures, potential risks and benefits, and their right to withdraw at any time without consequence. Informed consent was obtained electronically from all individual participants included in the study. To protect participant confidentiality, all data were collected and processed anonymously. No personally identifiable information was linked to the survey responses. Data were stored on password-protected secure servers, accessible only to the principal investigators for analysis purposes. Furthermore, the patient vignette (“David”) presented in the Attribution Questionnaire is a hypothetical, composite case. It was constructed for the purpose of this study and is not based on any identifiable individual.

## 3 Results

### 3.1 Descriptive statistics of participants

A total of 348 clinical nurses participated in this study. The sociodemographic and professional characteristics of the sample are presented in **Table 2**. Participants had a mean age of 40.40 years (SD = 8.64) and an average work experience of 16.57 years (SD = 8.62), indicating a sample of experienced nursing professionals. Male nurses accounted for 32.8% of the sample. The majority held a bachelor’s degree or higher (84.2%) and were married (80.2%). Regarding departmental distribution, 39.1% of the nurses worked in psychiatric departments, with the remainder in non-psychiatric departments.

**Table 2:** Sociodemographic and professional characteristics of participants (N = 348).

Variables	Categories	n	%	Mean	SD
Age (years)				40.40	8.64
Years of Experience				16.57	8.62
Gender	Male	114	32.8		
	Female	234	67.2		
Education Level	Bachelor’s or above	293	84.2		
	Associate degree or below	55	15.8		
Marital Status	Married	279	80.2		
	Unmarried/Other	69	19.8		
Department Affiliation	Psychiatric	136	39.1		
	Non-psychiatric	212	60.9		

### 3.2 Descriptive statistics of key variables

The scores for the main study variables are summarized in **Table 3**. The overall mean score for perceived stigma (PSPS) was 19.58 (SD = 3.70), indicating a moderate level. Among the attribution dimensions, personal responsibility (7.65 ± 1.54) and controllability (7.30 ± 1.83) scores were relatively high. Regarding emotional responses, the scores for sympathy (6.12 ± 2.59), fear (6.45 ± 2.17), and anger (5.99 ± 2.36) were similar. For behavior tendencies, the score for helping behavior tendencies (6.08 ± 2.11) was higher than for avoidance (5.27 ± 2.31), while the score for coercive intervention was the highest (7.27 ± 1.81).

**Table 3:** Scores of main study variables (N = 348, x ± s).

Variables/Subscales	Items	Scoring Range	Mean	SD
Perceived Stigma (PSPS Total)	9	9-45	19.58	3.70
Attribution (AQ)				
Personal Responsibility	2	2-18	7.65	1.54
Controllability	2	2-18	7.30	1.83
Emotional Response (AQ)				
Sympathy	3	3-27	6.12	2.59
Fear	3	3-27	6.45	2.17
Anger	3	3-27	5.99	2.36
Behavioral Tendency (AQ)				
Helping Behavior Tendencies	2	2-18	6.08	2.11
Avoidance	2	2-18	5.27	2.31
Coercion	2	2-18	7.27	1.81

### 3.3 Correlation analysis

Pearson correlation coefficients among the key variables are displayed in **Table 1**. As hypothesized, sympathy was significantly and positively correlated with helping behavior tendencies (r= .269, p<.05). A weak but significant positive correlation was found between perceived stigma and sympathy (r= .160, p<.05). The total attribution score was strongly correlated with emotional responses of sympathy (r= .416, p<.05), fear (r= .712, p<.05), and anger (r= .670, p<.05). Notably, the attribution of personal responsibility showed no significant correlation with helping behavior tendencies (r= .033, p>.05) but was positively correlated with coercive intervention (r= .433, p<.05).

### 3.4 Group comparisons between psychiatric and non-psychiatric nurses

Independent samples t-tests were conducted to compare psychiatric and non-psychiatric nurses on all study variables (see **Table 4**). Contrary to our initial hypothesis (H4), no statistically significant differences were found between the two groups in their levels of perceived stigma (t(344) = -0.227, p= .821) or sympathy (t(344) = 0.564, p= .573).

**Table 4:** Comparisons between psychiatric and non-psychiatric nurses on study variables (Independent samples t-tests).

Variable	Psychiatric nurses (n=136)	Non-psychiatric nurses (n=210)	t	df	p
	M	SD			
Perceived Stigma (PSPS)	19.51	3.45	19.60	3.87	-0.227
Sympathy	6.23	2.38	6.07	2.73	0.564
Dangerousness	5.04	1.60	5.56	1.71	-2.993
Fear	5.76	2.22	6.91	2.03	-4.97
Responsibility	7.35	1.54	7.37	1.54	-0.101
Anger	5.47	2.32	6.35	2.32	-3.433
Segregation	5.72	1.82	5.15	1.77	2.315
Perceived Treatability	5.54	1.85	5.43	1.74	0.575
Helping Behavior Tendencies	6.62	1.67	5.74	2.29	3.865
Avoidance	4.70	2.09	5.64	2.39	-3.77
Coercion	7.39	1.71	7.10	1.87	1.439
Attribution (QA Total Score)	56.21	9.05	57.71	9.63	-1.177

M, Mean; SD, Standard Deviation. Significant results (p <.05) are presented in bold.

However, significant differences emerged in other key dimensions. Compared to non-psychiatric nurses, psychiatric nurses perceived patients with AUDs as significantly less dangerous (t (344) = -2.993, p= .003) and reported significantly lower levels of fear (t (344) = -4.970, p<.001) and anger (t (344) = -3.433, p= .001). In terms of behavioral tendencies, psychiatric nurses demonstrated a significantly stronger intention to help (t(344) = 3.865, p<.001) and a significantly lower tendency to avoid​ patients (t(344) = -3.771, p<.001). A notable finding was that psychiatric nurses also reported a higher tendency towards supporting segregation (e.g., social or institutional separation) of patients (t(344) = 2.315, p= .021). No significant group differences were found in attributions of personal responsibility, perceived treatability, endorsement of coercion, or in the overall attribution score.

### 3.5 Mediation analysis of the relationship between perceived stigma and helping behavior tendencies via sympathy

**Figure 1:**
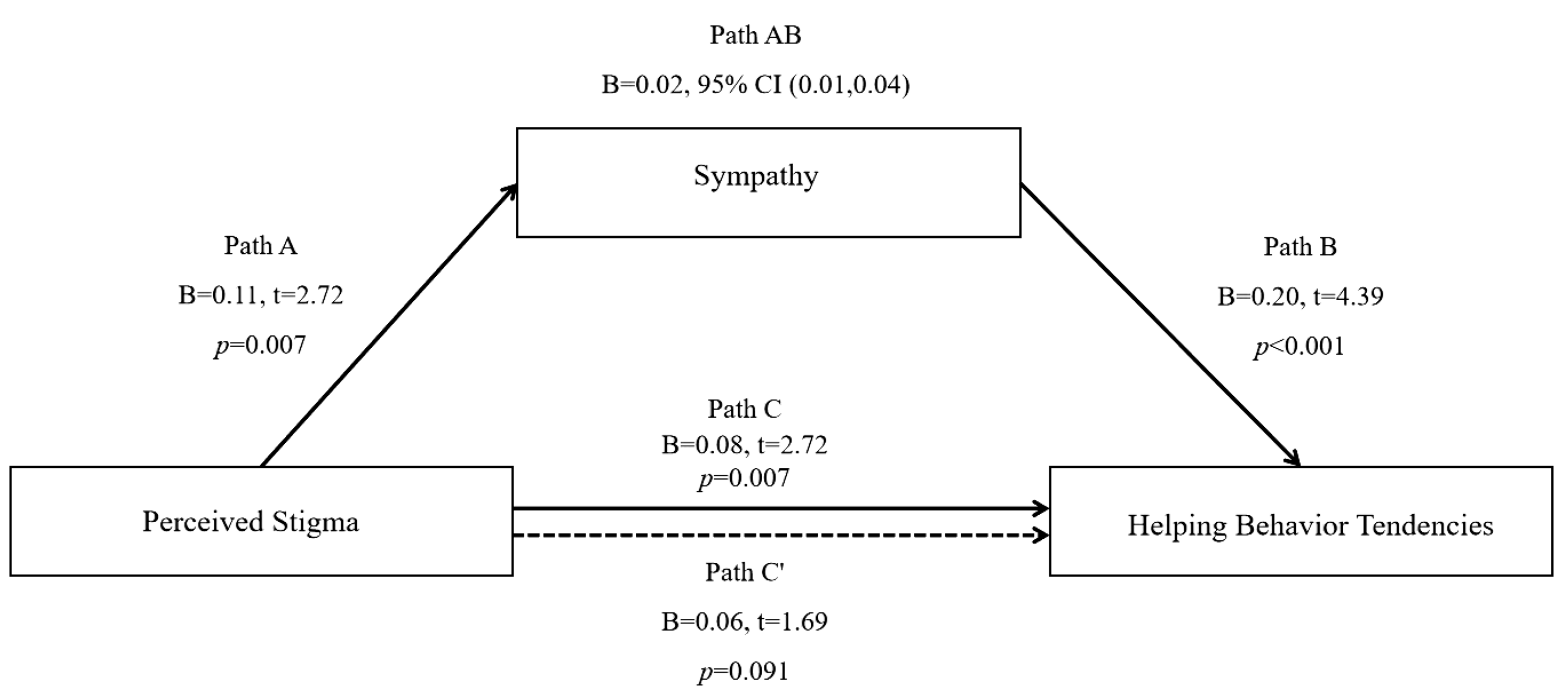
The mediating role of sympathy in the relationship between perceived stigma and helping behavior tendencies. Unstandardized path coefficients are presented. Solid lines indicate significant paths (p<.05), and the dashed line indicates a non-significant path (ns). The indirect effect was tested using 5,000 bootstrap samples.

To test the hypothesis that sympathy mediates the relationship between perceived stigma and helping behavior, a simple mediation model was examined using Hayes’ PROCESS macro (Model 4) with 5,000 bootstrap samples. The hypothesized model is depicted in **Figure 1**, with perceived stigma as the independent variable (X), sympathy as the mediator (M), and helping behavior tendencies as the dependent variable (Y).

As shown in **Figure 1**, the path coefficients were as follows: perceived stigma was significantly positively associated with sympathy (Path A: B = 0.11, t= 2.72, *p* = .007, 95% CI [0.03, 0.19]). Sympathy, in turn, was significantly positively associated with helping behavior tendencies (Path B: B = 0.20, t= 4.39, *p* <.001, 95% CI [0.11, 0.29]). The direct effect of perceived stigma on helping behavior tendencies, after accounting for the mediator, was not significant (Path C’: B = 0.06, t= 1.69, p= .091, 95% CI [-0.01, 0.13]).

The bootstrap analysis revealed a significant indirect effect of perceived stigma on helping behavior tendencies through sympathy. The unstandardized indirect effect estimate was 0.02, with a 95% bias-corrected confidence interval of [0.01, 0.04]. Since the confidence interval did not include zero and the direct effect (Path C’) was non-significant, the results are consistent with a model in which sympathy fully mediates the relationship between nurses’ perceived stigma and their helping behavior tendencies toward patients with AUDs. The mediation model accounts for the observed association entirely through this emotional pathway.

## 4 Discussion

### 4.1 Summary of key findings: a dual-pathway perspective

This study yielded three core findings. First, nurses reported moderate levels of perceived stigma toward patients with AUDs, consistent with its documented prevalence in healthcare (24, 25).This was accompanied by similarly moderate and complex emotional (sympathy, fear, anger) and behavioral responses, reflecting an ambivalent stance potentially stemming from the incomplete integration of the neurobiological model of addiction into practice (26) and internal conflict (27, 28). Second, and central to this study, sympathy was found to fully mediate the relationship between perceived stigma and helping behavior tendencies, revealing a novel “stigma-to-sympathy-to-helping” pathway. Third, significant differences between psychiatric and non-psychiatric nurses indicated that professional specialization reshapes the cognitive-affective-behavioral responses following stigma recognition.

### 4.2 Correlation and pathway: the dual role of stigma and the centrality of sympathy

Correlation analysis provided a foundation for a more nuanced understanding. While perceived stigma was only weakly correlated with helping behavior tendencies, it showed a significant positive relationship with sympathy. This key finding challenges the simplistic view of stigma as a direct barrier to care and aligns with the proposed dual-pathway model. When stigma is filtered through cognitive appraisal that incorporates neurobiological understanding, it can lead to reduced perceptions of dangerousness and controllability, and may thereby be associated with sympathy rather than solely negative emotions such as anger or fear (29–31).

The core discovery of this study is that sympathy fully mediated the relationship between perceived stigma and helping behavior tendencies. This means that stigma’s influence on behavior is not direct but is entirely channeled through the emotional response of sympathy. Nurses who perceive higher societal stigma, but who translate that perception into feelings of concern and sympathy for the patient, are more likely to engage in helping behavior tendencies. This underscores sympathy as a critical pivot point, linking a potentially negative social cognition (stigma) into a potential motivator for prosocial action (32–34). This pattern was distinct from other pathways, as fear and anger were linked to avoidance and coercion, respectively, further emphasizing the unique functional role of sympathy in promoting voluntary care. This mediating role of sympathy was distinct from the patterns observed for other negative emotions. Consistent with Weiner’s theory, anger and fear were positively correlated with tendencies for avoidance and coercive intervention (see **Table 1**), but they did not demonstrate a significant mediating link between perceived stigma and helping behavior tendencies. This alignment of negative emotions with distanced or controlling responses resonates with findings from the Chinese context, where attributional and emotional processes have similarly been identified as key determinants of social distance in stigma-related outcomes (35).This contrast underscores the unique function of sympathy in linking the awareness of societal stigma into a motivation for voluntary, supportive care, whereas anger and fear remain aligned with distancing or controlling responses.

### 4.3 The impact of professional background: specialized training reshapes responses

A pivotal finding was the significant difference between psychiatric and non-psychiatric nurses. Contrary to the hypothesis, there was no difference in overall perceived stigma or sympathy. This suggests that foundational stigma is pervasive across specialties (7, 36). However, psychiatric nurses reported significantly lower perceptions of patient dangerousness, lower levels of fear and anger, a stronger tendency to help, and a lower tendency to avoid. This profile indicates that specialized training does not necessarily erase the label of stigma but profoundly alters its psychological and behavioral sequelae (37, 38).

Training likely fosters more nuanced attributions, reducing the perception of threat (dangerousness) and the associated negative emotions (fear, anger) that are linked to avoidance (39–41). Consequently, the behavioral inclination shifts towards approach and help. The higher score on segregation among psychiatric nurses requires careful interpretation; it may not reflect a punitive social distance but rather a professional judgment favoring structured, therapeutic separation as part of treatment, distinct from rejection-based avoidance (42, 43). This pattern strongly advocates for the integration of addiction neuroscience and therapeutic communication skills into core nursing curricula to replicate these adaptive cognitive-emotional-behavioral patterns in general nurses (44–46).

### 4.4 Practical implications for nursing education and clinical intervention

Based on these findings, we propose concrete recommendations to inform nursing education and clinical intervention. First, mandatory education on the neurobiology of addiction should be embedded within nursing curricula to reframe AUDs from a moral failing to a chronic health condition (47–50). Second, professional training should move beyond fostering general empathy to specifically cultivate other-oriented emotions such as sympathy. Techniques like patient narrative immersion, perspective-taking exercises, and supervised clinical debriefing can strengthen the link between understanding patient vulnerability and fostering compassionate helping behavior tendencies (51, 52). Furthermore, structured clinical placements in addiction or psychiatric settings for all nursing students, coupled with mentorship from specialist nurses, can provide safe exposure to reduce fear and build practical, help-oriented competencies (53).

## 5 Limitations

This study has limitations. First, due to the cross-sectional design, the mediation analysis supports the hypothesized associations but cannot confirm causal directions or rule out alternative explanatory models. Second, self-reported measures may be subject to bias. Third, the use of a convenience sample may limit the generalizability of the findings. Fourth, as the survey link was disseminated through internal organizational channels, the exact number of nurses who received the invitation (the denominator) could not be determined; therefore, a traditional response rate cannot be calculated, which may limit the assessment of sample representativeness. Future research should use longitudinal or experimental designs and behavioral observations to verify causality. Expanding sampling across regions and settings would improve representativeness. Qualitative methods could further explore cognitive and contextual influences, while examining additional moderators or mediators would enrich the model.

## 6 Conclusion

This study reveals that the relationship between stigma and nursing care for individuals with AUDs is not direct but is critically mediated by emotional responses. Specifically, sympathy acts as a key mechanism that can channel the awareness of stigma into motivation for helping behavior tendencies. Furthermore, specialized psychiatric training is associated with a more adaptive response pattern characterized by reduced threat perception, diminished negative emotions, and increased helping behavior tendencies, even when the underlying recognition of stigma persists. These findings suggest a necessary shift in intervention strategies: from merely attempting to reduce stigma toward actively cultivating the specific cognitive reappraisals and emotional competencies that underpin compassionate and professional care for this patient population.

## Data Availability

The original contributions presented in the study are included in the article/**Supplementary Material**. Further inquiries can be directed to the corresponding authors.

## References

[1] Finn SW Mejldal A Nielsen AS . Perceived barriers to seeking treatment for alcohol use disorders among the general Danish population - a cross sectional study on the role of severity of alcohol use and gender. Arch Public Health. (2023) 81:65. doi: 10.1186/s13690-023-01085-4. PMID: 37087483 PMC10122805

[2] Koob GF . Alcohol use disorder treatment: Problems and solutions. Annu Rev Pharmacol Toxicol. (2024) 64:255–75. doi: 10.1146/annurev-pharmtox-031323-115847. PMID: 38261428

[3] Schomerus G Leonhard A Manthey J Morris J Neufeld M Kilian C . The stigma of alcohol-related liver disease and its impact on healthcare. J Hepatol. (2022) 77:516–24. doi: 10.1016/j.jhep.2022.04.026. PMID: 35526787

[4] Tyerman J Patovirta AL Celestini A . How stigma and discrimination influences nursing care of persons diagnosed with mental illness: A systematic review. Issues Ment Health Nurs. (2021) 42:153–63. doi: 10.1080/01612840.2020.1789788. PMID: 32762576

[5] Subu MA Wati DF Netrida N Priscilla V Dias JM Abraham MS . Types of stigma experienced by patients with mental illness and mental health nurses in Indonesia: a qualitative content analysis. Int J Ment Health Syst. (2021) 15:77. doi: 10.1186/s13033-021-00502-x. PMID: 34663399 PMC8524985

[6] Rigney N Hong W . Prosocial helping behavior: Conceptual issues and neural mechanisms. Biol Psychiatry. (2025) 97:961–70. doi: 10.1016/j.biopsych.2025.03.003. PMID: 40090565 PMC12358107

[7] Kruse EA Dodell-Feder D . Schizophrenia spectrum stigma in healthcare: a systematic review. Front Psychiatry. (2025) 16:1648957. doi: 10.31219/osf.io/qwvx2_v2 40927017 PMC12414995

[8] van Beukering IE Smits SJC Janssens KME Bogaers RI Joosen MCW Bakker M . In what ways does health related stigma affect sustainable employment and well-being at work? A systematic review. J Occup Rehabil. (2022) 32:365–79. doi: 10.1007/s10926-021-09998-z. PMID: 34487290 PMC9576674

[9] Weiner B . An attributional theory of achievement motivation and emotion. Psychol Rev. (1985) 92:548–73. doi: 10.1007/978-1-4612-4948-1_6. PMID: 3903815

[10] Weiner B Perry RP Magnusson J . An attributional analysis of reactions to stigmas. J Pers Soc Psychol. (1988) 55:738–48. doi: 10.1037/0022-3514.55.5.738. PMID: 2974883

[11] Volkow ND Koob GF McLellan AT . Neurobiologic advances from the brain disease model of addiction. N Engl J Med. (2016) 374:363–71. doi: 10.4324/9781003032762-5. PMID: 26816013 PMC6135257

[12] Volkow ND Koob G . Brain disease model of addiction: why is it so controversial? Lancet Psychiatry. (2015) 2:677–9. doi: 10.1016/s2215-0366(15)00236-9. PMID: 26249284 PMC4556943

[13] Buchman DZ Imahori D Lo C Hui K Walker C Shaw J . The influence of using novel predictive technologies on judgments of stigma, empathy, and compassion among healthcare professionals. AJOB Neurosci. (2024) 15:32–45. doi: 10.1080/21507740.2023.2225470. PMID: 37450417

[14] Gilbert P . Compassion: From its evolution to a psychotherapy. Front Psychol. (2020) 11:586161. doi: 10.3389/fpsyg.2020.586161. PMID: 33362650 PMC7762265

[15] Yitshaki R Kropp F Honig B . The role of compassion in shaping social entrepreneurs’ prosocial opportunity recognition. J Bus Ethics. (2022) 179:617–47. doi: 10.1007/s10551-021-04860-x. PMID: 34131355 PMC8192050

[16] Puhl RM . Weight stigma and barriers to effective obesity care. Gastroenterol Clin North Am. (2023) 52:417–28. doi: 10.1016/j.gtc.2023.02.002. PMID: 37197883

[17] Karimian A Sadooghiasl A Khoobi M Mohammadi E Kazemnejad A . Clinical nurses’ experiences of discrimination against patients: A qualitative study in Iran. West J Nurs Res. (2025) 47:1065–74. doi: 10.1177/01939459251352300. PMID: 40652332

[18] Kılıç-Demir B Kızılpınar S . Stigmatization of patients with mental disorders: a comparative study of nurses in forensic psychiatry and inpatient settings. Front Psychiatry. (2024) 15:1440917. doi: 10.3389/fpsyt.2024.1440917. PMID: 39211536 PMC11358071

[19] Baminiwatta A Alahakoon H Herath NC Kodithuwakku KM Nanayakkara T . Trait mindfulness, compassion, and stigma towards patients with mental illness: A study among nurses in Sri Lanka. Mindfulness (N Y). (2023) 14:979–91. doi: 10.1007/s12671-023-02108-5. PMID: 37090854 PMC10031165

[20] Chang CC Chang KC Hou WL Yen CF Lin CY Potenza MN . Measurement invariance and psychometric properties of perceived stigma toward people who use substances (PSPS) among three types of substance use disorders: Heroin, amphetamine, and alcohol. Drug Alcohol Depend. (2020) 216:108319. doi: 10.1016/j.drugalcdep.2020.108319. PMID: 33027709

[21] Luoma JB O’Hair AK Kohlenberg BS Hayes SC Fletcher L . The development and psychometric properties of a new measure of perceived stigma toward substance users. Subst Use Misuse. (2010) 45:47–57. doi: 10.3109/10826080902864712. PMID: 20025438 PMC5067154

[22] Corrigan PW Rowan D Green A Lundin R River P Uphoff-Wasowski K . Challenging two mental illness stigmas: personal responsibility and dangerousness. Schizophr Bull. (2002) 28:293–309. doi: 10.1093/oxfordjournals.schbul.a006939. PMID: 12693435

[23] Shrout PE Bolger N . Mediation in experimental and nonexperimental studies: new procedures and recommendations. Psychol Methods. (2002) 7:422–45. doi: 10.1037/1082-989x.7.4.422. PMID: 12530702

[24] Cazalis A Lambert L Auriacombe M . Stigmatization of people with addiction by health professionals: Current knowledge. A scoping review. Drug Alcohol Depend Rep. (2023) 9:100196. doi: 10.1016/j.dadr.2023.100196. PMID: 38023342 PMC10656222

[25] Parish CL Feaster DJ Pollack HA Horigian VE Wang X Jacobs P . Healthcare provider stigma toward patients with substance use disorders. Addiction. (2025) 120:2005–19. doi: 10.1111/add.70122. PMID: 40702596

[26] Frank LE Nagel SK . Addiction and moralization: the role of the underlying model of addiction. Neuroethics. (2017) 10:129–39. doi: 10.1007/s12152-017-9307-x. PMID: 28725284 PMC5486499

[27] O’Donnell A Kaner E Hanratty B Gilvarry E Wigham S Jackson K . Care professionals’ accounts of providing support and treatment for people with co-occurring alcohol use disorder and depression in the North East of England, UK: A qualitative study informed by complexity theory. PloS One. (2025) 20:e0334524. doi: 10.4324/9781003544883-6. PMID: 41091787 PMC12527159

[28] Merrick TT Louie E Cleary M Molloy L Baillie A Haber P . A systematic review of the perceptions and attitudes of mental health nurses towards alcohol and other drug use in mental health clients. Int J Ment Health Nurs. (2022) 31:1373–89. doi: 10.1111/inm.13043. PMID: 35909095 PMC9796325

[29] Galanis C Leske M Hamamura T Weber N Hing N Delfabbro PH . Stigma in substance-based and behavioural addictions: A systematic review. J Behav Addict. (2025) 14:79–99. doi: 10.1556/2006.2024.00072. PMID: 39819679 PMC11974440

[30] Sattler S Zolala F Baneshi MR Ghasemi J Amirzadeh Googhari S . Public stigma toward female and male opium and heroin users. An experimental test of attribution theory and the familiarity hypothesis. Front Public Health. (2021) 9:652876. doi: 10.3389/fpubh.2021.652876. PMID: 33959582 PMC8096178

[31] Pennington CR Monk RL Heim D Rose AK Gough T Clarke R . The labels and models used to describe problematic substance use impact discrete elements of stigma: A registered report. Psychol Addict Behav. (2023) 37:723–33. doi: 10.31234/osf.io/7trmf. PMID: 37166945

[32] Eisenberg N Eggum ND Di Giunta L . Empathy-related responding: Associations with prosocial behavior, aggression, and intergroup relations. Soc Issues Policy Rev. (2010) 4:143–80. doi: 10.1111/j.1751-2409.2010.01020.x. PMID: 21221410 PMC3017348

[33] Eisenberg N Fabes RA Miller PA Fultz J Shell R Mathy RM . Relation of sympathy and personal distress to prosocial behavior: a multimethod study. J Pers Soc Psychol. (1989) 57:55–66. doi: 10.1037//0022-3514.57.1.55. PMID: 2754604

[34] McCauley TG McAuliffe WHB McCullough ME . Does empathy promote helping by activating altruistic motivation or concern about social evaluation? A direct replication of Fultz et al. (1986). Emotion. (2024) 24:1868–84. doi: 10.31234/osf.io/7vsau. PMID: 38976420

[35] Zeng X . Somatized or stigma? Causal attributions and emotional responses in shaping social distance towards people with mental illness, China. Heliyon. (2024) 10:e32985. doi: 10.1016/j.heliyon.2024.e32985. PMID: 39021942 PMC11252714

[36] Oliveira AM MaChado D Fonseca JB Palha F Silva Moreira P Sousa N . Stigmatizing attitudes toward patients with psychiatric disorders among medical students and professionals. Front Psychiatry. (2020) 11:326. doi: 10.3389/fpsyt.2020.00326. PMID: 32425827 PMC7207477

[37] Wong JCM Chua JYX Chan PY Shorey S . Effectiveness of educational interventions in reducing the stigma of healthcare professionals and healthcare students towards mental illness: A systematic review and meta-analysis. J Adv Nurs. (2024) 80:4074–88. doi: 10.1111/jan.16127. PMID: 38402635

[38] Zhamaliyeva L Ablakimova N Batyrova A Veklenko G Grjibovski AM Kudaibergenova S . Interventions to reduce mental health stigma among health care professionals in primary health care: A systematic review and meta-analysis. Int J Environ Res Public Health. (2025) 22(9):1441. doi: 10.3390/ijerph22091441. PMID: 41007584 PMC12469428

[39] Santonja-Ayuso L Andreu-Pejó L Carmona-Simarro JV . Working on mental health stigma in education: a multicentre community-based clinical trial. Front Public Health. (2025) 13:1515444. doi: 10.3389/fpubh.2025.1515444. PMID: 40655223 PMC12248066

[40] Potts LC Bakolis I Deb T Lempp H Vince T Benbow Y . Anti-stigma training and positive changes in mental illness stigma outcomes in medical students in ten countries: a mediation analysis on pathways via empathy development and anxiety reduction. Soc Psychiatry Psychiatr Epidemiol. (2022) 57:1861–73. doi: 10.1007/s00127-022-02284-0. PMID: 35451604 PMC9375761

[41] Funk M Drew Bold N Muturi C Lazeri L Kalina O Martinez-Viciana C . Breaking stigma, discrimination and promoting rights: global evaluation of the World Health Organization QualityRights e-training on mental health, recovery and community inclusion. BJPsych Open. (2025) 11:e185. doi: 10.1192/bjo.2025.10779. PMID: 40820972 PMC12451731

[42] Daguman EI Taylor A Flowers M Lakeman R Hutchinson M . Differentiating therapeutic responses that reduce restrictive practice use and situational aggression in an acute mental health unit. J Clin Nurs. (2025) 34:4698–709. doi: 10.1111/jocn.17727. PMID: 40084814 PMC12489444

[43] Yersin G Silva B Golay P Morandi S . Mental health professionals’ perceptions and attitudes towards seclusion: The ambivalent relationship between safety and therapeutic considerations. Int J Law Psychiatry. (2024) 97:102033. doi: 10.1016/j.ijlp.2024.102033. PMID: 39426043

[44] Fox JM Wason K Beers D Faulds M Lincoln N Tomanovich M . The creation of an addiction nursing fellowship program for registered nurses: A unique approach to enhancing the addiction-treatment workforce. Subst Abus. (2023) 44:24–31. doi: 10.1177/08897077231169566. PMID: 37226903

[45] Compton P Blacher S . Nursing education in the midst of the opioid crisis. Pain Manag Nurs. (2020) 21:35–42. doi: 10.1016/j.pmn.2019.06.006. PMID: 31358464

[46] Semerci R Savaş EH . The effects of child health nursing curriculum-integrated therapeutic activities on therapeutic communication skills of nursing students: Non-randomized study. J Pediatr Nurs. (2023) 73:221–7. doi: 10.1016/j.pedn.2023.10.026. PMID: 37922859

[47] Newman LC Swisshelm AT . Teaching knowledge and empathy in substance use disorder through enriched education in the neurobiology of addiction: A narrative review on addiction education in professional schools. Subst Use Addctn J. (2025) 46:735–45. doi: 10.1177/29767342251317026. PMID: 39927518

[48] Volkow ND Boyle M . Neuroscience of addiction: Relevance to prevention and treatment. Am J Psychiatry. (2018) 175:729–40. doi: 10.1176/appi.ajp.2018.17101174. PMID: 29690790

[49] Rezapour T McLean KL Psederska E Maleki KN Ekhtiari H Vassileva J . Neuroscience-informed psychoeducation for addiction: a conceptual and feasibility study. Front Psychiatry. (2025) 16:1527828. doi: 10.3389/fpsyt.2025.1527828. PMID: 40012711 PMC11862476

[50] Kyzar EJ Arbuckle MR Abba-Aji A Balachandra K Cooper J Dela Cruz A . Leveraging neuroscience education to address stigma related to opioid use disorder in the community: a pilot study. Front Psychiatry. (2024) 15:1360356. doi: 10.3389/fpsyt.2024.1360356. PMID: 38563031 PMC10982477

[51] Byrne M Campos C Daly S Lok B Miles A . The current state of empathy, compassion and person-centred communication training in healthcare: An umbrella review. Patient Educ Couns. (2024) 119:108063. doi: 10.1016/j.pec.2023.108063. PMID: 38008647

[52] Nelson SW Germann C Yudkowsky R Pare B Wendell L Blackie M . Changing behavior and promoting clinical empathy through a patient experience curriculum for health profession students. AEM Educ Train. (2024) 8:e11048. doi: 10.1002/aet2.11048. PMID: 39600911 PMC11586136

[53] Moxham L Roberts M Yousiph T Jay EK Lewer K Robson G . ‘I can’t see myself seeking help’: The influence of clinical placements on nursing students’ stigmatising beliefs and intentions to seek help for their own mental health issues: A prospective cohort study. Int J Ment Health Nurs. (2025) 34:e13429. doi: 10.1111/inm.13429. PMID: 39302041 PMC11751763

