# Supplementary material for: From stigma to support: the mediating role of sympathy between nurses’ perceived stigma and helping behavior tendency for alcohol use disorder
Source: Front Psychiatry. 2026 Apr 22;17:1811126. doi: 10.3389/fpsyt.2026.1811126 (PMC13143928; doi:10.3389/fpsyt.2026.1811126)
Supplement: Supplementary file 1 [file DataSheet1.pdf]

Supplementary Material: Survey Instruments

Part 1: Your Background Information

Please provide the following information about yourself. All responses are anonymous and confidential.

1. What is your age? ______ years
2. How many years have you worked as a registered nurse? ______ years
3. What is your gender?

[ ] Male

[ ] Female

4. What is your highest educational qualification?

[ ] College diploma or below

[ ] Bachelor’s degree or above

5. What is your marital status?

[ ] Married

[ ] Unmarried/Other

6. Do you currently work in a psychiatric department/unit?

[ ] Yes

[ ] No

Part 2: Perceptions of Social Attitudes (PSPS)

Instruction: Please read each statement carefully. From 1 (Strongly Disagree) to 5 (Strongly Agree), choose the score that best represents your view—that is, your perception of how most people in society view or act towards individuals who have received treatment for alcohol use disorder.

No.	Statement	1 (Strongly Disagree)	2	3	4	5 (Strongly Agree)
1	Most people would be willing to be a close friend with someone who has received treatment for alcohol use disorder.					
2	Most people believe that someone who has been treated for alcohol use disorder is just as trustworthy as the average person.					
3	Most people would approve of someone who has received treatment for alcohol use disorder serving as a teacher.					
4	Most people would hire someone who has been treated for alcohol use disorder to babysit their children.					
5	Most people look down on someone who has received treatment for alcohol use disorder.					

6	Most employers would hire a qualified applicant who has been treated for alcohol use disorder.					
7	Most employers would pass over the application of a person treated for alcohol use disorder in favor of another applicant.					
8	Most people would be willing to date someone who has been treated for alcohol use disorder.					
9	Most people believe that having received treatment for alcohol use disorder is a shameful thing.					

Part 3: Reactions to a Specific Case (AQ)

Instruction: Please read the following description about David:

David is 32 years old, works as an office clerk, and lives with his parents. He has alcohol use disorder and has been hospitalized several times for this issue.

Note: This is a hypothetical, composite vignette created for research purposes and does not describe a real individual.

Based on the description above, please indicate the extent to which you agree with each of the following statements by selecting a number from 1 (Not at all)to 9 (Very much).

**No.**	**Statement**	**1 (Not at all)**	**2**	**3**	**4**	**5**	**6**	**7**	**8**	**9 (Very much)**
1	I feel pity for David.									
2	How dangerous do you think David is?									
3	How scared do you feel of David?									
4	To what extent is David himself responsible for his current condition?									
5	How angry would you feel at David?									
6	I think it would be best for David’s family if he were put away in a psychiatric hospital.									
7	I think that David’s condition can be greatly improved with treatment.									
8	How likely is it that you would help David?									
9	I would try to stay away from David.									
10	How much do you agree that David should be forced into treatment with his doctor even if he does not want to?									
